# Comparative Analysis of Diagnostic Techniques for *Helicobacter pylori* Infection: Insights for Effective Therapy

**DOI:** 10.1111/jcmm.70487

**Published:** 2025-03-19

**Authors:** Ahmed Mujtaba, Muhammad Suhail Ibrahim, Sana Parveen, Noreen Sarwar, Suliman A. Alsagaby, Muhammad Ahsan Raza, Mohamed A. Abdelgawad, Mohammed M. Ghoneim, Ahmed H. El‐Ghorab, Samy Selim, Waleed Al Abdulmonem, Muzzamal Hussain, Tadesse Fenta Yehuala

**Affiliations:** ^1^ Department of Food Science and Technology, Faculty of Engineering Sciences and Technology Hamdard University Islamabad Campus Islamabad Pakistan; ^2^ Institute of Food and Nutritional Sciences PMAS‐Arid Agriculture University Rawalpindi Pakistan; ^3^ Institute of Microbiology University of Veterinary and Animal Sciences Lahore Pakistan; ^4^ Department of Medical Laboratory Sciences, College of Applied Medical Sciences Majmaah University AL‐Majmaah Saudi Arabia; ^5^ Department of Zoology Govt. Graduate College for Boys Gujranwala Pakistan; ^6^ Department of Pharmaceutical Chemistry, College of Pharmacy Jouf University Sakaka Aljouf Saudi Arabia; ^7^ Department of Pharmacy Practice, College of Pharmacy AlMaarefa University Riyadh Saudi Arabia; ^8^ Department of Chemistry, College of Science Jouf University Sakaka Saudi Arabia; ^9^ Department of Clinical Laboratory Sciences, College of Applied Medical Sciences Jouf University Sakaka Saudi Arabia; ^10^ Department of Pathology, College of Medicine Qassim University Buraidah Kingdom of Saudi Arabia; ^11^ Department of Food Science Government College University Faisalabad Faisalabad Pakistan; ^12^ Faculty of Chemical and Food Engineering, Bahir Dar Institute of Technology Bahir Dar University Bahir Dar City Ethiopia

**Keywords:** comparative analysis, diagnostic test, *H. pylori*, patients, sensitivity, specificity

## Abstract

Effective therapy against *Helicobacter pylori* hinges on a timely and accurate diagnosis. The objective is to assess *H. pylori* infection in dyspeptic patients and compare various indicative tests. After approval, gastrointestinal biopsies and blood samples of 96 subjects exhibiting gastroduodenal symptoms were collected; both invasive and non‐invasive tests were employed to analyse the samples. Results revealed 40 cases (41.67%) positive for *H. pylori* via histopathology and rapid urease testing, while 46 subjects tested positive for IgA and IgG antibodies via ELISA. Eighteen biopsies showed positivity in the culture test, corroborated by endoscopic examination and biochemical assessments (urease, catalase and oxidase). The isolates showed various degrees of resistance to antibiotics, while polymyxin B showed the highest (100%) followed by amoxicillin (88.90%) and kanamycin (77.78%). Additionally, the *CagA* gene presence was detected in 18 individuals through molecular methods. Sensitivity and specificity percentages (%) varied among diagnostic methods: histopathology (95/77), rapid urease (100/83.5), gram staining (85.7/90), IgG serology (100/66.6), IgA serology (100/79.5), PCR (100/75), RUT and IgG serology combination (100/79.04), and RUT, Gram staining and IgG serology combination (100/92.4), respectively. PCR emerged as the most reliable test. In the current investigation, other tests also exhibited high sensitivity and specificity values. Thus, employing comparative detection methods rather than relying solely on one methodology is advisable for accurate detection.

## 1 Introduction

*Helicobacter pylori* is characterised as a spiral‐shaped, Gram‐negative, microaerophilic bacterium, having uni‐polar motile sheathed flagella and belonging to the order *Campylobacterales* ([1, 2]). Colonisation of the bacterium is usually linked with the advancement of peptic gastritis of the gastrointestinal tract, which may lead to ulceration and gastric lymphoma [3]. Approximately half of all global stomach infections are attributed to *H. pylori* . Since detection and isolation by Marshall and Warren in Australia in 1982, the bacterium has been associated as a causative agent of chronic gastritis [2]. Subsequently, the International Agency for Research on Cancer (IARC) designated the bacterium as a carcinogen, founded on comprehensive epidemiological and histological data [4].

Typically, the acquisition of the bacterium occurs early in life and persists throughout one's lifetime. The current literature suggests that the spread of disease occurs from human to human, either through the gastric oral route and faecal‐oral route or through oral contact [5]. While food and water cannot be ruled out as potential sources of infection, as they are not the primary means of transmission; on the other side, alcohol and tobacco use, blood type (ABO), genetic predisposition, and overall health status have also been investigated, but findings have been inconsistent [6].

Diagnosing bacterial infections can be challenging, yet it is crucial for effective treatment and management. Various diagnostic tests are employed to isolate and characterise the disease‐causing bacteria. Invasive tests like endoscopic examinations with biopsy collection are commonly utilised for histology, rapid urease, culture, and other tests. Conversely, non‐invasive methods include serological examinations, stool antigen tests, molecular tests and urea breath tests (UBT) [7]. However, each method has its limitations. Culture and biopsy identification require expertise, while serology may not distinguish between active and past infections. UBT offers high sensitivity and specificity but can yield false positives in the presence of other urease‐producing bacteria. Additionally, it demands specialised equipment and may yield positive results in culture‐negative subjects [8]. Culturing and identifying the bacterium in biopsy samples demands proficiency and skill due to its fastidious nature, while the rapid urease test and microscopy can be highly specific when conducted correctly, as they depend on biopsy samples, which can be prone to sampling error, similar to culture [9]. Serological tests are preferred in certain clinical scenarios where local stomach changes might influence results during the course of therapy. Though locally developed serological (ELISA) tests tend to offer superior specificity, sensitivity and accuracy compared to available ones. Nonetheless, serology struggles to differentiate between active, asymptomatic and past infections, especially in children, and cannot discern the disease. Carbon‐labelled urea breath tests exhibit superior specificity and sensitivity compared to counterparts, yet their specificity may decrease due to urease production by various organisms. Moreover, they necessitate specific instruments and reagents and are prone to show false positive results even in culture‐negative patients. Detection through polymerase chain reaction (PCR), with its heightened sensitivity and specificity, is commonly employed for infection analysis; however, due to genomic variability among strains, targeted gene identification in tested samples may not always be successful [10, 11, 12].

The precision in commonly utilised techniques for detection of infection is consistently evaluated in medical contexts. Moreover, a comprehensive report comparing the isolation frequency in various regions and the effectiveness of current diagnostic methods for identifying and characterising the disease is still lacking. Therefore, this study was undertaken to investigate subject with dyspepsia for potential *Helicobacter pylori* contagion and to relate the efficacy of different frequently employed indicative tests.

## 2 Materials and Methods

### 2.1 Participants and Design

Overall, 440 consecutive patients, comprising males aged 35 to 50 and females aged 35 to 55, were approached at various local hospitals in Rawalpindi and Islamabad to participate in the study. Among them, 129 patients met the specified inclusion and exclusion criteria. However, 33 of these patients were either discontinued from care or lost during follow‐up. Consequently, a final cohort of 96 patients (with a 1:1 gender ratio) was included in the study, following doctors' recommendations for clinical indications requiring endoscopy at clinic. Exclusion criteria were based on guidelines outlined earlier by our study [13]. The study group comprised patients not using antibiotics, anti‐secretary drugs, nonsteroidal anti‐inflammatory drugs, bismuth or corticosteroids and excluding of pregnant and lactating mother. Each patient received detailed information regarding the study's purpose, procedures and expected outcomes, and written consent was obtained prior to their participation. All study techniques involving patients were approved by the Ethical Approval Committee of PMAS‐Arid Agriculture University on 2 February 2017.

### 2.2 Biopsy and Blood Sample Collection

The participants underwent endoscopic examination through an Olympus Tokyo, Japan, video endoscope to assess any visible lesions on the mucosa of the upper gastrointestinal tract. Tissue samples were collected from the antrum and corpus and stored in sterile saline at 4°C. These samples were promptly transported to the laboratory within 1 h for further analysis [14].

A sample of 3–5 mL blood was collected prior to biopsy or any therapy, and the sample was brought to room temperature. Following centrifugation (Spectrafuge TM, USA) at 1000 g for 10 min, these specimens were placed into a sterile Eppendorf tube. The resultant serum was stored in a lig freezer (DW‐45, China) at −20°C until further examination.

### 2.3 Histopathological Observations

This test was conducted following the method outlined by Dixon et al. [15], with certain modifications. Gastric biopsies were initially fixed in a 10% solution of formalin for 1 day and then embedded in paraffin. Tissue sections were then cut at 0.3 μm and underwent hydration in descending grades of alcohol after paraffin removal, followed by cutting into chronological 4‐μm diameters. One section was stained with eosin and haematoxylin, while another was stained with Giemsa to reveal the presence of *H. pylori* . The bacterium was identified as curved rods on the surface of gastric epithelial cells.

### 2.4 Bacteria Culture

Samples of biopsy underwent manual grinding to achieve uniform clusters, then were streaked onto tryptic soy agar (Merck & Co., USA) with the addition of 2% agar and sheep blood (5%). Subsequently, the plates were incubated at 37°C for 3 to 5 days in microaerophilic Gas‐Pak jars (BBL Microbiology System, MD, USA) with envelopes.

### 2.5 Morphology of Colonies

The colony's physical traits, such as shape, surface and colour, were examined with a magnifying glass once the colonies appeared on the plates, following streaking and a subsequent incubation at 37°C for 3 to 5 days.

### 2.6 Gram Staining

A small portion of a colony was collected and placed on a glass slide having sterilised distilled water. After that, the heat fixation of the smear was done over a flame. Subsequently, the glass slide was marked with crystal violet for 60 s and then rinsed with tap water. After that, iodine solution was applied to the slide for 30 to 60 s and rinsed with tap water. The ethyl alcohol (95%) was poured until the crystal violet colour was completely gone. After 1 min of water flushing, the smear was counterstained for 1 min with safranin. Ultimately, the slides were cleaned and patted dry before being examined with a microscope [16].

### 2.7 Hydrogen Peroxidase Analysis

The catalase enzyme production was assessed following the procedure of Berg et al. [17]. Bacterial colonies were transferred onto a slide using a sterile loop, then covered with H_2_O_2_ (3%) solution, and the presence of bubbles was observed as a positive result.

### 2.8 Oxidase Test

N, N, N, N‐tetramethyl‐p‐phenylenediaminedihydrochloride (1%) was dissolved in deionised and sterilized water, and bacterial culture was introduced by using a loop. At last, the detection of a colour shift, particularly the appearance of a blue hue, was observed to indicate successful outcomes [17].

### 2.9 Rapid Urease Test

Gastric biopsies were utilised to test the urease activity of *H. pylori* in gastric mucosal specimens by observing colour change (pH shifts) resulting from the breakdown of the urea into ammonia by urease. The process was performed using the Helicotech UT Plus kit (Strong Biotech Corporation, Taiwan). Results were interpreted using the Helicotech UT Plus within 5 min to 1 h; a yellow colour indicated a negative result, while a shift to pink or magenta signified a positive result.

### 2.10 Urease Test

The urease test was performed according to the protocol done by [18]. To prepare the culture, phenol red (0.02 g) was combined with 10% urea and pH was adjusted to 6.8 ± 1. The mixture was then sterilised by autoclaving (15 psi, 15 min and 121°C). Subsequently, the broth was inoculated with a loopful of the organism culture and placed in an incubator for 1 h at 37°C for 1 h. The conversion of colour from yellow to pink indicated the existence of bacteria.

### 2.11 Antibiotic Resistance

Antibiotics susceptibility testing of 18 isolates was done using the *E*‐test method [19, 20]. Initially, colonies were supplemented with 7% sheep blood in 10 mL broth (Thermofischer Scientific, USA) until reaching a turbidity equivalent to the three Mac Farland standards. The suspension was then swabbed onto a selective agar plate (150 mm diameter). Following drying, antibiotic strips were put on the plates, which were then incubated in a microaerophilic environment at 37°C for 3 to 5 days using a BBL Microbiology System generator envelope (USA). The percentage of inhibition at the site where the inhibition zone intersected the strip was measured and compared with a control strain (ATCC 43504). The antibiotic susceptibility was tested for amoxicillin (> 2 μg/mL), polymyxin (300 UNITs), tetracycline (4 μg/mL), kanamycin (30 μg/mL), rifampin (5 μg/mL), clarithromycin (2 μg/mL) and trimethoprim (2.5 μg/mL) (MASTDISCS, Bioanlyse), respectively.

### 2.12 ELISA

Serum specimens underwent testing for antibody IgG/IgA with NovaLisa TM enzyme‐linked immunosorbent assay kit (Nova Tec Immundignosica GmbH) following the manufacturer's guidelines. Results exceeding 30 NTU/mL were deemed positive.

### 2.13 DNA Extraction for Polymerase Chain Reaction

The NaCl 100 mM, tris–HCl 10 mM and sodium dodecyl sulphate 0.5% buffer solution was used for the collection of gastric biopsy samples. The samples were kept in a freezer at −70°C until further analysis. Subsequent to digestion using the buffer of lysozyme, DNA extraction was carried out employing cetyltrimethyl ammonium bromide. According to Abu‐Sbeih et al. [14], mixtures were cultured at 37°C for 1 day (IMC 18, Thermo Fischer Scientific, US). Stringent measures were taken during sample collection and preparation to prevent any potential contamination.

### 2.14 Polymerase Chain Reaction Analysis

Briefly, 2 μL of DNA template was supplemented to a mixture comprising 18 μL, including 10 pmol each of *CagA*‐F (5′ AATACACCAACGCCTCCAAG‐3′) and *CagA*‐R (5′‐TTGTTGCCGCTTGCTCTC‐3′) primers, 1X Polymerase chain reaction buffer, comprising KCl (50 mM), triton X‐100 (1.5%), tris–HCl at pH 8.3 (10 mM), in addition to 200 mM of d NTPs, 1.5 mM of Mgcl_2_ and 1 U of Taq polymerase, respectively. This mixture underwent PCR amplification using a Mastercycler X50 Cycler (Eppendorf, Germany) according to the following program: initial denaturation for 5 min at 96°C, 40 cycles of denaturation at 94°C for 1 min, annealing at 62°C for 1 min and elongation at 72°C for 2 min. At last, a 10‐min extension step was performed to ensure complete extension of the product. After that, gel electrophoresis (ET‐H1iobase, China) was performed [having 1X tris acetate buffer (100 mL) under 70 and 110 V and 110 mA for 20 to 30 min] in a gel stained with 1% ethidium bromide and visualised under an ultraviolet trans‐illuminator (Slimeline TM, Spectronics Corporation, USA) for the presence of amplified DNA.

### 2.15 Statistical Analysis

The obtained data were analysed in triplicates and the means were tested for one‐way ANOVA using software (Statistix 8.1) as described by Steel et al. [21] with a confidence interval of 95%. The tests efficacy was measured in percentages (%) with the following formulas:

$$\text{Sensitivity}=\left[\left\{\text{true positive}/\left(\text{true positive}+\text{false negative}\right)\right\}\right]\times 100$$

$$\text{Specificity}=\left[\left\{\text{true negative}/\left(\text{false positive}+\text{true negative}\right)\right\}\right]\times 100$$

$$\text{Positive predictive value}=\left[\left\{\text{true positive}/\left(\text{false negative}+\text{true negative}\right)\right\}\right]\times 100$$

$$\text{Negative predictive value}=\left[\left\{\text{true negative}/\left(\text{true negative}+\text{false negative}\right)\right\}\right]\times 100$$

$$\text{Accuracy}=\left[\left(\text{true negative}+\text{true positive}\right)/\left(\text{true negative}+\text{true positive}+\text{false negative}+\text{false positive}\right)\right]\times 100$$

## 3 Result

### 3.1 Data Collection Analysis

At the outset, 440 individuals displaying potential signs of infection from different hospitals were examined. The study's aims were explained, and individuals were actively encouraged to take part in the study. The complete case history form was completed by collecting demographic information, such as age, gender, blood type, and address, as well as infection symptoms (such as fullness, epigastric pain, heartburn, etc.), as well as the individual's medical history, including any previous illnesses and medications. Based on predefined exclusion criteria, 344 subjects (78.2%) were deemed ineligible for the study. These excluded participants comprised 15 breastfeeding mothers (4.4%), 136 individuals previously using NSAIDs, antibiotics and bismuth drugs (39.5%), 160 who were not willing to take part in research (46.5%) and 33 others (9.6%t) who had not come to the clinic or were not available for follow‐up.

Analysis of the case history data indicated an equal distribution of genders among the 96 subjects, with 48 individuals of each gender. These subjects were aged between 35 and 55 years, with a mean age of 47 years. Around 40 patients (41.7%) reported enduring stomach pain persistently for 6 to 8 months, while 24 individuals (25%) reported experiencing heartburn. Furthermore, two patients (8.3%) had a documented past of peptic ulceration (refer to Table 1).

**TABLE 1:** Gastric biopsy samples, medical history, rapid urease test, endoscopic, histopathological and serology tests results of patients.

	Positive, 40 (41.67%)	Negative, 56 (58.33%)	Total, 96 (100)		Test	Patients (%)
Stomach/Abdominal pain	16 (40%)	24 (42.86%)	40 (41.67%)	Endoscopic findings	Gastritis	62 (64.58%)
Heart Burn	8 (20%)	16 (28.57%)	24 (25%)		Gastroesophageal reflux	16 (16.67%)
Sex: Male/Female	24 (60%)/16 (40%)	24 (43%)/32 (57%)	48 (50%)/48 (50%)		Duodenitis	10 (10.42%)
Mean Age (yr)	52	41	47		Peptic and duodenal ulceration	8 (8.33%)
Range	50–55	35–49	35–55	Histopathology	Positive	41 (42.71%)
History of peptic ulcer	0 (0)	2 (14.28%)	2 (8.33%)		Negative	55 (57.29%)
				RUT	Positive	40 (41.67%)
					Negative	56 (58.33%)
					**Serology test**	
					**Total**	**Positive *N* (%)**
				IgG	96	46 (47.92%)
				IgA	96	40 (41.67%)
				Control IgG	20	8 (40%)
				Control IgA	20	6 (30%)

### 3.2 Histopathology, Endoscopic Examination and Rapid Urease Test

**FIGURE 1:**
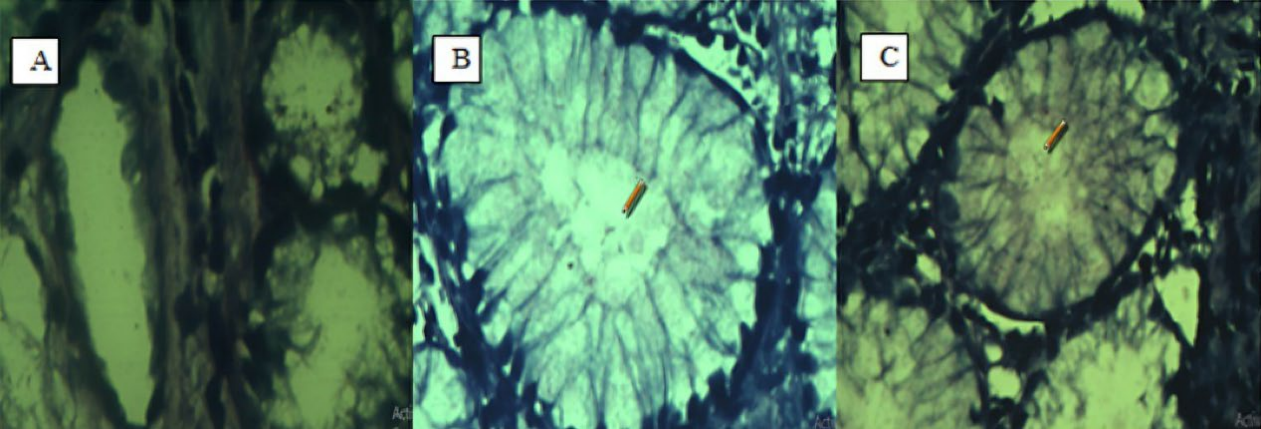
Histopathology examination results. (A) Negative histopathological result in gastric gland; there is no bacteria and no inflammation. (B, C) Positive histopathological results showed the presence of *Helicobacter pylori*.

Rapid urease tests (RUTs) and histopathology were performed on 96 patients to determine whether they were infected with *H. pylori*. The results exhibited that 41.67% of subjects (*n* = 56) tested positive for RUTs, while 58.33% (*n* = 41) tested negative. Conversely, 41 patients (42.71%) were histopathologically positive, while 55 patients (57.29%) tested negative (see Figure 1). Endoscopic evaluations showed that 62 patients (64.58%) had signs of gastritis, 16 patients (16.67%) were diagnosed with gastroesophageal reflux, 10 patients (10.42%) had duodenitis, and 8 patients (8.3%) exhibited duodenal or peptic ulceration.

### 3.3 Serum Enzyme Linked Immunosorbent Test

**FIGURE 2:**
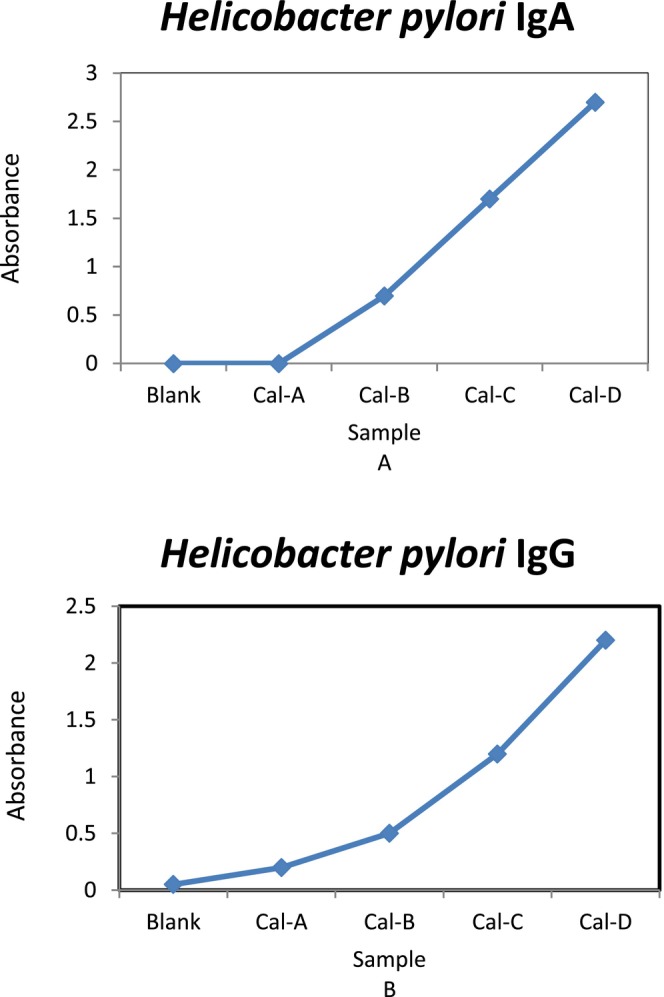
The standard curve of the ELISA standard and blank solution anti‐ *H. pylori* IgA and IgG.

The 96 patients under focus were tested for IgA and IgG antibodies against *H. pylori* . In 40 (41.67%) patients, anti‐*H. pylori* IgA antibodies were detected, while in 46 (47.91%) patients, anti‐*H. pylori* IgG antibodies were identified. ELISA reports rely significantly on the measurement of optical density, which results from the interface among antibodies and the substrate result, leading to the development of a yellow colour. Table 1 summarises the findings of concentration and optical density of anti‐ *H. pylori* . A curve is also shown in Figure 2 depicting the effects of anti*‐H. pylori* IgA and IgG antibody optical density and concentration for 96 models.

### 3.4 Microscopic Examination, Oxidase, Urease and Catalase Test

From a total of 96 biopsy specimens, *n* = 18 *isolates* of *H. pylori* were isolated. These isolates were cultivated on tryptic soy agar enhanced with sheep blood in the micro‐aerophilic environment for 3–5 days. Initially, the isolates were characterised based on colony morphological traits, appearing as transparent with limited growth and presenting elongated, spiral‐shaped specimens that stained gram‐negative. Additionally, biochemical examinations including oxidase, catalase and the urease tests were conducted on all detected bacterial colonies (*n* = 18), which tested positive for bacteria. The results indicated that all isolates exhibited transparent, spiral forms and tested positive for the oxidase test. Among the various combinations of tests, the highest positivity rate was detected in the urease test combined with serology (40), followed by the combination of the urease test with gram stain or culture (18) and the urease test with gram staining (see Table 2).

**TABLE 2:** Morphological characteristics and confirmatory tests.

	Positive	Negative
Morphological characteristics		
Transparent colonies	18 (18.75%)	NG
Long and spiral shape	18 (18.75%)	NG
Confirmatory test		
Gram staining	24 (25%)	72 (75%)
Oxidase	20 (20.83%)	76 (79.17%)
Catalase	40 (41.67%)	56 (58.33%)
Urease	43 (44.79%)	53 (55.21%)
Culture	18 (18.75%)	78 (81.25%)
Urease test + gram staining	18 (18.75%)	78 (81.25%)
Urease test + serology	40 (41.67%)	56 (58.33%)
Urease test + gramstaining + serology	16 (16.67%)	80 (83.33%)

Abbreviation: NG, no growth.

### 3.5 Antibiotic Susceptibility Test

The 18 isolates that tested positive in biochemical assays were evaluated for their vulnerability to different antibiotics (see Table 4). Predominant resistance patterns were observed: 100% of strains were resistant to polymyxin B (*n*‐18) whereas 88.89% of strains were resistant to amoxicillin (*n* = 16), 77.78% of strains were resistant to kanamycin (*n* = 14), 22.2% of strains were resistant to clarithromycin (*n* = 4), 16.7% of strains were resistant to tetracycline (*n* = 3), 11.1% of strains were resistant to trimethoprim (*n* = 2) and 5.6% of strains were resistant to rifampin (*n* = 1). Additionally, the outcomes indicated sensitivity rates for rifampin, trimethoprim, tetracycline, clarithromycin and kanamycin of 94.4%, 88.9%, 83.3%, 66.7% and 22.2%, respectively (Table 3).

**TABLE 3:** Antibiotic susceptibility testing of 18 strains of *Helicobacter pylori*.

Antibiotics	Resistance *N* (%)	Intermediate *N* (%)	Sensitive *N* (%)
Amoxicillin (> 2 μg/mL)	16 (88.90%)	2 (11.10%)	—
Clarithromycin (2 μg/mL)	4 (22.22%)	2 (11.11%)	12 (66.67%)
Kanamycin (30 μg/mL)	14 (77.78%)	—	4 (22.22%)
Polymyxin (B300UNITs)	18 (100%)	—	—
Rifampin (5 μg/mL)	1 (5.56%)	—	17 (94.44%)
Tetracycline (4 μg/mL)	3 (16.67%)	—	15 (83.33%)
Trimethoprim (2.5 μg/mL)	2 (11.11%)	—	16 (88.89%)

### 3.6 Molecular Detection of Isolates

**FIGURE 3:**
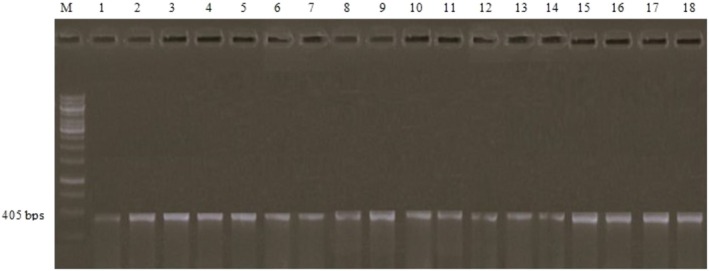
The amplified *CagA* gene for biopsy specimens on agarose gel (1%) electrophoresis of PCR amplification of the *CagA* gene frequent fragment lane.

The PCR techniques were employed to validate the outcomes of conventional diagnostic approaches (such as ELISA, RUT, histopathology and many other biological assessments). The 18 isolates were genotyped, and their DNA concentrations were determined. Genomic DNA was isolated from the isolates; the *CagA* gene was amplified with specific primers and detected afterward. DNA amplified from the strains showed a band of about 405 bp in size (refer to Figure 3). Details of the examination and histopathology study for the total 18 specimens are outlined in Table 4.

**TABLE 4:** Patient data subjected to molecular test and test result.

No	Sex	Age	Biochemical test						ELISA (NTU/mL)		Endoscopic examination
			Histo.	Culture	Oxidase	Catalase	Urease	RUT	IgG	IgA	
13	M	38	+	+	+	+	+	+	32	31.5	Gastritis
15	M	45	+	+	+	+	+	+	45	38.7	Gastric ulcer
19	F	40	+	+	+	+	+	+	35	32.4	Acute duodenal ulcer
24	M	55	+	+	+	+	+	+	31	36	Gastritis
29	F	55	+	+	+	+	+	+	30.3	33.1	Acute duodenal ulcer
31	F	50	+	+	+	+	+	+	35	38	Gastritis
32	M	48	+	+	+	+	+	+	38	32	Gastritis
38	F	40	+	+	+	+	+	+	104	38	Gastric ulcer
40	M	42	+	+	+	+	+	+	42	30.2	Gastritis
42	M	48	+	+	+	+	+	+	41	35	Gastritis
44	M	53	+	+	+	+	+	+	32.5	33	Acute duodenal ulcer
45	M	52	+	+	+	+	+	+	60.8	31.1	Gastritis
60	F	39	+	+	+	+	+	+	35	30.2	Gastritis
62	F	41	+	+	+	+	+	+	130	64.7	Gastric ulcer
71	M	42	+	+	+	+	+	+	97	41.7	Gastric ulcer
75	F	35	+	+	+	+	+	+	43	32	*H. pylori* gastritis
88	F	38	+	+	+	+	+	+	88	40	Gastric ulcer
96	F	40	+	+	+	+	+	+	36.2	31.7	Gastritis

### 3.7 Statistical Analysis

The overall sensitivity, specificity, predictive value (both positive and negative), and accuracy of biological and molecular tests for *H. pylori* were evaluated and are detailed in Table 5. The rapid urease test, IgA and IgG serology, polymerase chain reaction, as well as combinations such as RUT + IgG serology and RUT + gram staining + IgG serology, showed significantly higher sensitivity percentages (100%, *p* > 0.05). Conversely, the highest specificity values were observed with combinations like RUT + gram staining and IgG serology (92.4%), followed by gram staining alone (90%). The highest positive and negative predictive values were found as 81.2% and 94.0% for histopathology, 70.32% and 90.20% for IgA serology, and 78.10% and 99.0% for the RUT + gram staining + IgG serology, respectively. Notably, the highest accuracy was achieved with the polymerase chain reaction test (94.20%), and then the RUT + gram staining + IgG serology combination (94.0%) and gram staining alone (89.0%).

**TABLE 5:** Statistical analysis of various diagnostic tests for *Helicobacter pylori*.

Test	Sensitivity (%)	Specificity (%)	PPV (%)	NPV (%)	Accuracy (%)
Histology	95.0A	77.0C	81.2A	94.0AB	86.0F
RUT	100A	83.5B	61.0CD	100A	81.0D
Gram staining	85.7B	90.0A	66.0BC	96.0AB	89.0B
IgG serology	100A	66.6D	59.0D	98.0AB	77.0E
IgA serology	100A	79.5BC	70.2B	90.2B	80.4D
PCR	100A	75.0C	66.7BC	100A	94.2A
RUT + IgG serology	100A	79.04BC	68.0B	100A	85.1C
RUT + gram staining + Ig G serology	100A	92.4A	78.1A	99.0AB	94.0A
*p*	0.0002	0.0000	0.0000	0.0012	0.0000

## 4 Discussion

The pattern and occurrence of ailment exhibit considerable variation both among and within countries, with the highest infection rates often associated with densely populated areas and lower socioeconomic status. While global efforts to implement safety measures have contributed to a decline in disease incidence [22], its prevalence remains substantial, estimated at up to 50% in developed countries [23]. However, the occurrence is even higher in underdeveloping countries, accounting for approximately 90% [24]. The frequency of the *H. pylori* infestation increases with age worldwide [25]. Our study revealed a similar trend among Pakistani patients, consistent with findings from a study conducted in a low socioeconomic region of Sudan [26]. Additionally, our study found a higher prevalence of infection among males compared to females, as indicated in Table 1; these differences are in line with the observations reported by Lansdorp‐Vogelaar and Sharp [27]. A significant gender disparity was observed in a large cross‐sectional examination [28], which found a lower occurrence of *H. pylori* infection in females relative to males. This discrepancy in prevalence between genders may be attributed to differences in hygiene practices or social behaviours between females and males.

The results of the current study showed that 41.7% of indicative patients tested positive for *H. pylori* presence, which is similar to the findings of Abu‐Sbeih et al. [14], who identified a low isolation rate of *H. pylori* infection (53.7%) in Jordan. The isolation frequencies of *H. pylori* in the subcontinent are lower compared to global data. This is due to the organism's fastidious nature, which requires microaerophilic conditions, as well as the frequent use of antibiotics, including bismuth‐based drugs and the prevalence of protozoan infections in the region. Multiple factors such as age, ethnicity, geographic location, socioeconomic status, smoking habits, gender, transportation issues, delay in sample collection, insufficient tissue preparation, and uneven distribution of the causative agent in the gastric mucosa played a role in compromising the detection sensitivity and isolation of the organism [29]. Primarily, *H. pylori* infection is linked with various gastrointestinal symptoms such as chronic gastritis, metaplasia of the intestine and ulcer diseases. This was in agreement with the study of Conteduca et al. [30], who found similar results, indicating that persistent superficial gastritis caused by *H. pylori* increases the risk for gastric ulcers, atrophic gastritis, gastric cancer, mucosa‐associated lymphoid tissue lymphoma and gastric adenocarcinoma.

In any clinical setting, the demand for quick, accurate and economical methods for managing infections is desirable. Various techniques are available for diagnosing *H. pylori* infection [31], including bacteriological culture, histology examination, urease test, enzyme‐linked immunosorbent assay, PCR and antigen stool test, all of which are routinely used in medical identification. Subjects with concerning symptoms often undergo endoscopy for diagnosing *H. pylori* infection. In such cases, histopathology is commonly considered the primary diagnostic method for patients presenting with upper stomach symptoms or residing in regions with a notable prevalence of such cases. Histopathology enables the quantification of *H. pylori* and offers insights into the type and severity of inflammation in the stomach mucosa [32]. However, accurate histopathological investigation of gastritis by *H. pylori* depends heavily on the skills and experience of the examiner, as inter‐observer discrepancies have been reported in many studies. In our study, a pathologist often identified more helpful results when other analyses were negative, resulting in the specificity and sensitivity and importance of the pathologist's expertise. Urease examination is most widely utilised by investigators due to its rapid and cost‐effective nature, offering 79% to 100% sensitivity and 92% to 100% specificity [33]. However, false negative results may occur in urease testing because of irregular distribution of the bacterium, patients with active or recent bleeding, or the use of proton pump inhibitors or antibiotics [34]. In our study, we observed only a slight difference in accuracy between histopathology and the urease test. Therefore, the rapid urease test may be recommended as a gold standard method, as it provides results within an hour [35].

The basic objective of the current study was to compare non‐invasive and invasive methods for the diagnosis of infection. The data showed that 41 (42.71%) subjects tested positive for *H. pylori* via histopathology, while 40 subjects (41.67%) tested positive using the RUT method. Histology and RUT exhibited sensitivities of 95.0% and 100% for detecting *H. pylori* , and specificities of 77.0% and 83.5%, respectively. These findings are in line with those stated by Yuan et al. [36], who documented RUT results of 98.2%, 99.0%, 97.9% and 98.5% for sensitivity, specificity, positive predictive value, and negative predictive value, as well as accuracy, respectively. In this examination, histology demonstrated a sensitivity of 95.0% and specificity of 77.0%, which closely aligns with the findings of Khalifehgholi et al. [33]. It is worth noting that sensitivity and specificity values can vary dependent on the clinical context, the density of bacterial colonisation, and, to some extent, the practice and expertise of the researchers.

However, the *H. pylori* serologic test offers an affordable, user‐friendly solution widely employed for diagnosing infections in patients prior to treatment. Although in *H. pylori* antibody testing, sensitivity and specificity can range from 60% to 100% using recognised commercial laboratory‐based methods. The findings of the serological technique in the present study showed that 41.67% of the samples (*n* = 40) had anti *H. pylori* IgA, and 47.92% (*n* = 46) had IgG Anti *H. pylori* antibodies. These findings align with previously reported references, indicating the accuracy of the tests. The reliability of the serological test depends on the presence of *H. pylori* infection. To prevent misunderstanding, evaluation should be performed separately for each age category, emphasising not only the confirmed *H. pylori* status but also the presence of atrophic gastritis [37]. The sensitivity of the serology test was found to be 10%, consistent with the findings of Mohsun and Al‐Hadithi [38]. In the current study, the specificity of serology was counted to be 79.5% and 66.6% for anti IgA and IgG, which closely resembles the findings of Abu‐Sbeih et al. [14] at 82% and 60%, while Mahmood and Hamid [39] also reported a similar values for specificity. Serology serves as an accessible, sensitive, inexpensive and non‐invasive procedure for run. However, in this research, enzyme‐linked immunosorbent assay exhibited the lowest specificity and accuracy compared to other tests. The overall lower accuracy of the serology test can be attributed to its inability to differentiate current and past infections. Even though the information form did not include any participants who had received neither prior treatment, nor those who had occasionally recovered from *H. pylori* , test results could be falsely positive if there is a possibility of infections [11]. Furthermore, even after the infection has been eradicated, antibody titers can last for months, which may lead to false positive results [40].

Culture is commonly regarded as the gold standard for diagnosing microorganisms; however, several drawbacks have been noted in the case of *H. pylori* . These include the fact that it takes a long time to grow, that it is challenging to cultivate because of the generally low bacterial abundance, that it requires strict growth conditions, that it may infect biopsy forceps, that the bacterium is distributed unevenly on the stomach mucosa, and that it loses viability [14]. In the current study, *H. pylori* was detected positively in 18 samples. The obtained results were similar to Pilli and Kirani [41] findings, who reported 14% culture‐positive. The result pertaining to urease showed 44.79% positivity as compared to others, which might be due to recent or active bleeding or taking antibiotics. In addition, Pilli and Kirani [41] and Mohsun and Al‐Hadithi [38] also concluded positivity rates of 36% and 35.6%, respectively, for the urease test. Detection of causative agents can also be achieved using Gram staining. According to our study, there was a 25% positivity rate, which is close to the 22.2% positivity rate reported by Subbukesavaraja and Balan [42]. Gram staining exhibited a sensitivity of 85.7% and specificity of 90.0%, which is in line with the reported value of 80% for the specificity of gram staining.

Molecular approaches are being widely used for diagnosis because they offer rapid results, high sensitivity and specificity, and have minimal impact on the transportation system. Various PCR‐based techniques have been used to detect *H. pylori* [43]. There are several genes that have been targeted in these assays, including *CagA* and *ureA* genes, *UreC*, 16S rRNA and 26KDa species‐specific antigens (SSA) [33]. In this study, PCR was used to identify the presence of the *CagA* gene, which is a virulence factor. This test exhibited a 100% sensitivity and 75% specificity. Similar findings were also presented by Smith et al. [44] when PCR was used to identify the *CagA* gene. Conversely, Lu et al. [45] explored various PCR tests for *H. pylori* identification and found that *CagA* strengthening is highly specific but less sensitive compared to the detection of *UreC*, the 26 KDa species‐specific antigen gene and the 16S rRNA gene. The inconsistency may be ascribed to contamination of the sample by PCR products or inadequate endoscope disinfection. PCR methods play a crucial role in understanding *H. pylori* pathogenicity and immunisation. PCR is considered to be the important but highly sensitive procedure for organism detection and is particularly valuable for eradication assessment. The molecular techniques also facilitate the identification of pathogen‐related genes and antibacterial mutations, which contribute to the detection of microorganisms [46].

Based on the established results of the research, the accuracy of the tests, both individually and in combination, can be ranked as follows: PCR was ranked higher than RUT + gram staining + IgG serology than gram staining >histology > RUT + IgG serology>RUT > serology (PCR figures of Figure S1). This ranking may differ slightly across many researches. Nonetheless, a consistent trend is the preference for invasive methods over non‐invasive ones in nearly all studies. Furthermore, the gold standard cannot be determined by any of these methods alone. Moreover, this study suggests that combining tests yields higher sensitivity and specificity. It is advised that invasive and non‐invasive tests should be performed simultaneously for infection diagnosis.

Evaluating eradication to the sensitivity treatment is crucial due to the absence of universally successful regimens. Therefore, sensitivity testing should be conducted periodically and nationwide before initiating any treatment. Treatment failure in combating the disease can stem from various factors, but antimicrobial drug resistance stands out as the primary cause [47]. While susceptibility to antibiotics may vary between different regions, it is affected by prior use of these medications. Differences in antibiotic prescribing practices, community use of antibiotics, and mass eradication programs as part of prevention strategies may contribute to variations in susceptibility and resistance patterns across different regions. The efficacy of eradication therapy is likely to be impacted by these factors [48].

Testing for antibiotic susceptibility is advised when second‐line therapy fails, but it is not usually done for the diagnosis of a disease [43]. In this research, all 18 isolates exhibited varying sensitivity to the degrees of antibiotics commonly used as first‐line treatments, namely clarithromycin, trimethoprim, rifampicin and tetracycline. The result indicated that the strains are fully resistant to polymyxin and 88.90% resistant to amoxicillin. Otth and Wilson [49] and Mujtaba et al. [13] also reported similar findings in their prior studies. Additionally, the current outcomes underscore the critical need for establishing a resistance monitoring and surveillance system to mitigate treatment failures or the spread of resistance among the found strains.

## 5 Conclusion

There is limited evidence supporting the effectiveness of the tests used to diagnose *H. pylori* in Primary healthcare settings. In the current study, the accuracy of PCR tests (94.2%) is significantly higher in comparison to other tests like 89% gram staining, 86% histology, 81% RUT and 80% serology, as well as RUT + gram staining + IgG serology (94%) and RUT + serology (85.1%). PCR's sensitivity, specificity and accuracy made it seem like the most trustworthy method. Although invasive methods are often chosen and preferred over non‐invasive methods, many scientists do not consider any single invasive technique to be the gold standard due to many reasons. Additionally, the combination of diagnostic tests had a higher sensitivity and specificity as a result of the current investigation. Therefore, it is recommended to use both invasive and non‐invasive tests concurrently to confirm *H. pylori* disease. Using this at the primary healthcare level would help concentrate on available resources and generate future diagnostic evidence for the stratification of infection in a high‐throughput manner.

## Author Contributions

**Ahmed Mujtaba:** formal analysis (equal), investigation (equal), writing – original draft (equal). **Muhammad Suhail Ibrahim:** methodology (equal), supervision (equal), writing – review and editing (equal). **Sana Parveen:** conceptualization (equal), writing – review and editing (equal). **Noreen Sarwar:** data curation (equal), investigation (equal), writing – review and editing (equal). **Suliman A. Alsagaby:** resources (equal), writing – review and editing (equal). **Muhammad Ahsan Raza:** conceptualization (equal), data curation (equal), formal analysis (equal). **Mohamed A. Abdelgawad:** methodology (equal), writing – original draft (equal), writing – review and editing (equal). **Mohammed M. Ghoneim:** formal analysis (equal), resources (equal), writing – review and editing (equal). **Ahmed H. El‐Ghorab:** investigation (equal), project administration (equal), writing – review and editing (equal). **Samy Selim:** conceptualization (equal), methodology (equal), writing – review and editing (equal). **Waleed Al Abdulmonem:** conceptualization (equal), methodology (equal), writing – review and editing (equal). **Muzzamal Hussain:** conceptualization (equal), formal analysis (equal), methodology (equal), supervision (equal). **Tadesse Fenta Yehuala:** conceptualization (equal), data curation (equal), formal analysis (equal), writing – original draft (equal).

## Ethics Statement

The Ethics Committee of the Institute of Food and Nutritional Sciences at PMAS‐Arid Agriculture University, Rawalpindi, awarded us a grant for this study on 2 February 2017. The letter of ethical approval from the committee is attached to this document.

## Conflicts of Interest

The authors declare no conflicts of interest.

## Supporting information


Figure S1

## Data Availability

The data that support the findings of this study are available from the corresponding author upon reasonable request.
