# Supplementary figures and images for: Comparative Analysis of Diagnostic Techniques for Helicobacter pylori Infection: Insights for Effective Therapy
Source: J Cell Mol Med. 2025 Mar 19;29(6):e70487. doi: 10.1111/jcmm.70487 (PMC11921466; doi:10.1111/jcmm.70487)

**Supplementary File**

Figure 1: PCR pictures

| M | 1 | 2 | 3 | 4 | 5 | 6 | 7 | 8 | 9 | 10 | 11 | 12 | 13 | 14 | 15 | 16 | 17 | 18 |
| --- | --- | --- | --- | --- | --- | --- | --- | --- | --- | --- | --- | --- | --- | --- | --- | --- | --- | --- |


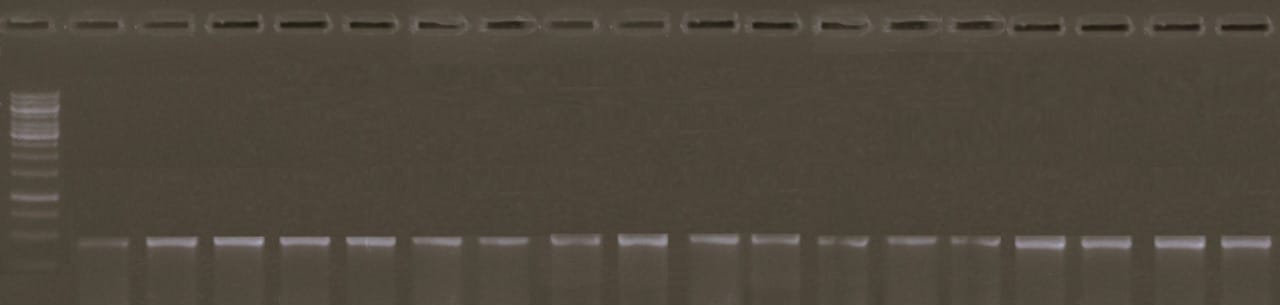


405 bps


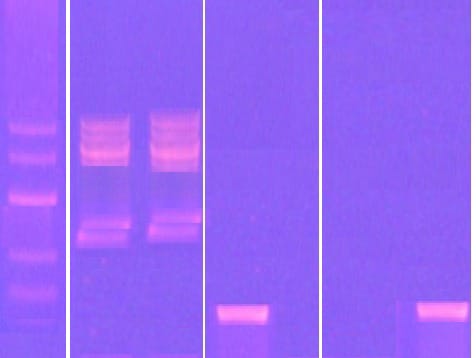


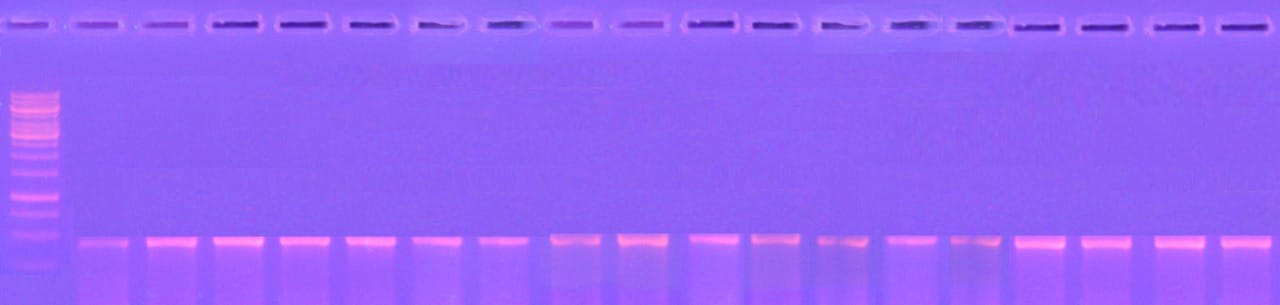


| M | 1 | 2 | 3 | 4 | 5 | 6 | 7 | 8 | 9 | 10 | 11 | 12 | 13 | 14 | 15 | 16 | 17 | 18 |
| --- | --- | --- | --- | --- | --- | --- | --- | --- | --- | --- | --- | --- | --- | --- | --- | --- | --- | --- |

Supplement: Supplementary file 1 — Figure S1 [file JCMM-29-e70487-s001.docx]
